# Bluetongue Risk Map for Vaccination and Surveillance Strategies in India

**DOI:** 10.3390/pathogens13070590

**Published:** 2024-07-16

**Authors:** Mohammed Mudassar Chanda, Bethan V. Purse, Luigi Sedda, David Benz, Minakshi Prasad, Yella Narasimha Reddy, Krishnamohan Reddy Yarabolu, S. M. Byregowda, Simon Carpenter, Gaya Prasad, David John Rogers

**Affiliations:** 1 ICAR-National Institute of Veterinary Epidemiology and Disease Informatics (NIVEDI), Ramagondanahalli, Yelahanka, Bengaluru 560064, India; 2 UK Centre for Ecology & Hydrology, Maclean Building, Crowmarsh Gifford, Wallingford OX10 8BB, UK; beth@ceh.ac.uk; 3 Lancaster Ecology and Epidemiology Group, Lancaster Medical School, Lancaster University, Furness Building, Lancaster LA1 4YG, UK; l.sedda@lancaster.ac.uk; 4 Department of Biology, University of Oxford, 11A Mansfield Road, Oxford OX1 3SZ, UK; david.benz@biology.ox.ac.uk (D.B.); david.rogers@biology.ox.ac.uk (D.J.R.); 5 National Research Centre on Equines, Sirsa Road, Hisar 125001, India; minakshi.abt@gmail.com; 6 Department of Animal Biotechnology, P.V. Narsimha Rao Telangana University, Hyderabad 500030, India; yellanarasimhareddy@gmail.com; 7 Vaccine Research Centre-Viral Vaccines, Centre for Animal Health Studies Tamil Nadu Veterinary and Animal Sciences University, Chennai 600051, India; drykmreddy@hotmail.com; 8 Institute of Animal Health and Veterinary Biological, Bengaluru 560024, India; byregowda@yahoo.com; 9 School of the Biological Sciences, 17 Mill Lane, Cambridge CB2 1RX, UK; stc46@cam.ac.uk; 10 International Institute of Veterinary Education & Research, Rohtak 124001, India; gprasad1986@gmail.com

**Keywords:** *Culicoides*, arbovirus, *Culicoides imicola*, remote sensing, risk mapping

## Abstract

Bluetongue virus (BTV, *Sedoreoviridae*: *Orbivirus*) causes an economically important disease, namely, bluetongue (BT), in domestic and wild ruminants worldwide. BTV is endemic to South India and has occurred with varying severity every year since the virus was first reported in 1963. BT can cause high morbidity and mortality to sheep flocks in this region, resulting in serious economic losses to subsistence farmers, with impacts on food security. The epidemiology of BTV in South India is complex, characterized by an unusually wide diversity of susceptible ruminant hosts, multiple vector species biting midges (*Culicoides* spp., Diptera: Ceratopogonidae), which have been implicated in the transmission of BTV and numerous co-circulating virus serotypes and strains. BT presence data (1997–2011) for South India were obtained from multiple sources to develop a presence/absence model for the disease. A non-linear discriminant analysis (NLDA) was carried out using temporal Fourier transformed variables that were remotely sensed as potential predictors of BT distribution. Predictive performance was then characterized using a range of different accuracy statistics (sensitivity, specificity, and Kappa). The top ten variables selected to explain BT distribution were primarily thermal metrics (land surface temperature, i.e., LST, and middle infrared, i.e., MIR) and a measure of plant photosynthetic activity (the Normalized Difference Vegetation Index, i.e., NDVI). A model that used pseudo-absence points, with three presence and absence clusters each, outperformed the model that used only the recorded absence points and showed high correspondence with past BTV outbreaks. The resulting risk maps may be suitable for informing disease managers concerned with vaccination, prevention, and control of BT in high-risk areas and for planning future state-wide vector and virus surveillance activities.

## 1. Introduction

Bluetongue virus (BTV) is the prototype virus of the *Orbivirus* genus in the *Sedoreoviridae* family [1]. Bluetongue is an economically important arbovirus that infects all wild and domestic ruminants and causes the disease bluetongue (BT), particularly in sheep. In India, BT is primarily observed in the southern states of Tamil Nadu, Karnataka, Telangana, and Andhra Pradesh [2], where it has a major impact on sheep production, particularly in subsistence farming where there is limited access to vaccines for livestock. BTV is biologically transmitted by biting midges of the genus *Culicoides* (Diptera: Ceratopogonidae), and at least seven putative vectors in India have been identified, primarily from studies carried out in other countries. In addition, at least 21 serotypes of BTV with limited cross-protection [3] and multiple ruminant species capable of being infected with the virus are known, resulting in one of the most complex epidemiology scenarios for BTV in the world.

Predictions of the presence and absence of BT outbreaks using logistic regression and climate, host, and land use factors are employed at the district level in India as part of the National Animal Disease Referral Expert System (NADRES) for early warning. There are more than eighty thousand villages classified in three states of South India to which BTV is known to be endemic (Andhra Pradesh, Karnataka, and Tamil Nadu), and villages in proximity differ substantially in the severity of BT outbreaks. Understanding the environmental conditions suitable for the presence and absence of BT at the village level is essential for effective systematic vaccination, the reduction of vector populations, and future surveillance.

Understanding the relationship between the presence and absence of the disease and its vectors, hosts, and environmental variables is important in defining risk areas and predicting incursions into new areas [4]. Climate data from weather stations have been used to construct models with spatial and temporal predictions of many vector-borne diseases, including BT [5,6,7]. More recently, there has been an increase in the use of MODIS data and other satellite data as predictor variables for risk mapping of infectious diseases [8,9,10], including BT [11,12]. Weather station data have a coarser spatial resolution than remotely sensed data, but weather stations directly measure certain parameters like air temperature, air humidity, and precipitation, which have a direct impact on the life history of different *Culicoides* species. However, the weather station data rarely reflect the microhabitats used by adult insects [13]. Due to this, the presence of only a few weather monitoring stations can hamper the quantification of relationships between vector and virus distribution and weather station-detected environmental variables. This effect has been demonstrated in comparisons between remote sensing data and weather station data in distribution models of *Culicoides imicola* [14].

South India contains several major vectors of BTV, including *C. imicola* Kieffer, *Culicoides brevitarsis* Kieffer, and *Culicoides oxystoma* Kieffer [15,16]. The habitat requirements for each species vary, and in the absence of species’ distribution data, it is difficult to understand the role of different environmental variables in determining their abundance, which ultimately causes outbreaks in South India. A village that reports outbreaks is considered an “epidemiological unit”, and each village that is affected is considered an outbreak. The number of cases in each village is also recorded. Most (60 out of 80) of the constituent districts of South India have reported BT outbreaks [2], and the fundamental vector, virus, and host parameters driving variation in the occurrence and severity of BT outbreaks remain unexplained.

Attempts have been made globally to model the incidence of vectors, particularly *C. imicola*, which has the widest distribution of any confirmed vector species. A logistic regression model for *C. imicola* in the Iberian Peninsula using averaged climate data was extrapolated to provide a distribution of the vector across the entire Mediterranean Basin [7]. There are also studies [6] in which averaged climate data were used, and a logistic regression approach was followed to study the effect of climate on the presence of *C. imicola* in Italy. The study obtained an overall 75% correct classification, but there was evidence of some misclassification of suitable *C. imicola* habitat due to the geographical clustering of sampling sites. A combination of the Maximum Entropy approach (Maxent), Boosted Regression Tree (BRT) and Ecological Niche Factor Analysis (ENFA) were used to develop a predictive spatial model for the presence of *Culicoides* species, and it was found that there was a positive correlation with precipitation variables and livestock densities and a negative correlation with altitude and temperature indices [17]. The current niche and future risk of *C. imicola* have also been modeled using a dynamic bioclimatic niche model (CLIMEX), and it was found that artificial irrigation played a key role in supporting the distribution of the BTV vector worldwide [18].

Remotely sensed variables have been used as both environmental classifications and surrogates of meteorological variables in spatial and temporal models of many vector-borne diseases [19,20]. These approaches are usually open-access and cover much broader geographical areas in more detail than meteorological station records do. There are several studies reporting NDVI (Normalized Difference Vegetation Index) as one of the important predictors in the models of BTV vector distribution and abundance [21,22]. When the models were used to predict the distribution of different vector species using remotely sensed variables, different sets of variables were selected as there are differences in the life history requirements of different *Culicoides* species [13,23].

The presence and absence of a host, virus, or vector is often determined not only by environmental variables (remotely sensed or weather station data) but also by seasonality. Seasonality in tropical and subtropical countries, such as India, is primarily defined by rainfall, in contrast to temperate and sub-temperate regions where temperature is more important. Using unprocessed time series data (monthly remotely sensed variables or weather station data) is not advised for reflecting seasonality due to serial correlation in the data. Principal component analysis (PCA) [24] is the most common technique in data ordination methods, but seasonality is lost in this approach [25]. Temporal Fourier analysis (TFA) of remotely sensed variables [26] overcomes the problem of serial correlation and helps capture the seasonality in the environmental conditions. While collinearity may still be present in datasets, TFA can be used to independently predict discriminating variables that drive seasonal pathogen transmission. The use of TFA imagery and its advantages have been discussed in mapping different vector-borne diseases [25], *Culicoides* species, and BT [22].

There is a limited number of veterinary personnel involved in animal disease surveillance and vaccination [27]. The Department of Animal Husbandry and Dairying and the state’s Animal Husbandry Department supply vaccines to farmers against important livestock diseases (e.g., Foot and Mouth Disease, Peste des Petits Ruminants (PPR), Haemorrhagic septicaemia, and others). The vaccines are manufactured by Indian Immunologicals, which falls under the National Dairy Development Board (NDDB). There are also state-run vaccine manufacturing units supported by state governments (e.g., the Institute of Animal Health & Veterinary Biologicals, Bengaluru, Karnataka), and these are mandated to fulfill the vaccination requirement for the entire state. The center provides monetary support to procure vaccines for diseases under control programs for the states. Initially, vaccines were provided at subsidized costs, but due to poor rates of uptake, the vaccines are now provided free of cost (including the vaccine costs and services of veterinarians). The vaccination programs are compulsory to implement under certain disease control programs, and the veterinarians are given performance targets based on the total susceptible population vaccinated in that area (https://dahd.nic.in/schemes/programmes/nadcp accessed on 5 June 2024). To date, the uptake of vaccination of ruminants with an available pentavalent vaccine for BTV that induces immunity against the most common serotypes in South India remains very limited.

In endemic countries, where multiple species of *Culicoides* may be involved in the transmission of BTV and a wide range of environmental factors may influence this process, there is a possibility of the presence of more than one epizootiological system. Therefore, it is important to understand the role of environmental variables in discriminating between the different systems. In this work, temporal Fourier processed variables that are remotely sensed are used to produce risk maps for BT by employing a non-linear discriminant analysis approach (NLDA) applied to BT presence and absence data in three South Indian states with the following objectives:

1. To identify the variables that discriminate between the presence and absence of BT outbreaks in South India.
2. To investigate whether environmental conditions in South India drive variation between the presence and absence of bluetongue outbreaks (more than one presence or absence group).
3. To test whether known but sparse absence data, or pseudo-absence data, give better accuracy in BT presence/absence models.
4. To develop a BTV risk map for South India to help in surveillance and vaccination strategies.

## 2. Materials and Methods

### 2.1. Bluetongue Presence and Absence Data

BT presence and absence data were collected from three states with different reporting systems. The BT data are a mix of clinical and laboratory confirmed cases using PCR, serological assays, and virus isolation. The data for Karnataka were obtained from the State Animal Disease Monitoring and Surveillance, Bangalore. The data for Andhra Pradesh were obtained from the State Department of Animal Husbandry, Hyderabad. The data for Tamil Nadu were obtained by visiting different districts where BTV was known to be endemic according to historical records (Tirunelveli, Madurai, Karur, Dindigul, and Erode). For other districts in Tamil Nadu, the data were collected from the Central Referral Laboratory (CRL), Chennai. There was no information on the diagnostic tests performed from the records of Tamil Nadu (both from CRL and during visits to the districts). All presence records for bluetongue were obtained for 1997 to 2011. No village-level records could be found before 1997.

The records for Karnataka and Tamil Nadu contained only the names of disease-affected villages, while the records for Andhra Pradesh contained the number of cases in each village. Only simple presence and absence records were considered in this analysis for all three states. The village names were first matched with the village databases for all three states, and the village centroids were then extracted for all the presence and absence records from the village-level shape files (obtained from the Survey of India through an individual license). There were 769 villages in all three states of South India that recorded BTV presence between 1997 and 2011 and 59,809 villages that did not report BT.

A village might also not have records of BT outbreaks because it is in fact disease-free (because conditions there are unsuitable for BTV, i.e., a genuine absence site) or because outbreaks have occurred there but have not been reported for various reasons (a genuine presence site but not recorded in the database). Because there are a variety of reasons for the records of BTV absence in the village database, such as lack of reporting, fewer number of veterinary personnel involved in reporting the disease, and lack of sensitive diagnostics in earlier years, the presence/absence models were run twice, once using the database-recorded village absence sites and once using ‘pseudo-absence’ data generated by a relatively standardized method in the eRiskMapper software employed [4,20].

Pseudo-absence points can be generated by many presence/absence statistical packages because most databases of animal or plant species or diseases fail to record genuine absences. In eRiskMapper, the user is allowed to define both a minimum and a maximum distance from any presence site where pseudo-absence sites might be randomly selected. The minimum distance attempts to guarantee that no pseudo-absence site is so similar in environmental conditions to a genuine presence site that it might actually be a suitable site for the species under study. The maximum distance attempts to guarantee that pseudo-absence sites are not so different from any presence site that their data are unable to distinguish local presence and absence. In the present models, the minimum distance was set to 0.5 degrees of latitude/longitude (approximately 50–60 km from the equator) and the maximum to ten degrees. However, the mask used to select pseudo-absence sites was restricted to the three South Indian states and the adjoining states (Kerala, some parts of Maharashtra, and Orissa).

### 2.2. Remotely Sensed Variables

In total, 50 temporal Fourier transformed MODIS variables (at 1 km spatial resolution) were used in the analysis [26]. The temporal Fourier processing extracts the seasonal information of the remotely sensed variables and describes it in terms of the mean, the annual minimum and maximum, the amplitudes and phases of the annual (A1 and P1), biannual (A2 and P2), and tri-annual (A3 and P3) components of the signal and the variance, respectively. The TF transformed MODIS variables were MIR (middle infrared), the daytime land surface temperature (dLST), the nighttime land surface temperature (nLST), the NDVI (Normalized Difference Vegetation Index), and the EVI (Enhanced Vegetation Index). The NDVI is a measure of photosynthetically active radiation (PAR) and has often been interpreted as an indicator, directly or indirectly, of chlorophyll abundance, vegetation biomass, soil moisture, and rainfall [28]. MIR is correlated with water content, surface temperature, and the structure of vegetation canopies, especially in young forest re-growth stands [29].

### 2.3. Non-Linear Discriminant Analysis Model (NLDA) Description

Non-linear discriminant analysis was carried out using a Windows-based eRiskMapper package based on the previous (non-Windows-based) software of Rogers [30].

Discriminant analysis is rather different from several other methods [31,32,33] used to describe species distributions because it is a classification method. The algorithm assigns each observation to a category in a mutually exclusive set of groups that encompass the entire range of variability expected.

#### 2.3.1. NLDA and Clustering

One of the assumptions of linear discriminant analysis is that all groups have the same covariance. This is usually violated for species’ distributions, and it is necessary to adapt the equations to allow for different covariance of the different groups (presence and absence in the simplest case). For two groups, this is shown in Equation (1), where C_i_ is the group-specific rather than common covariance matrix and D_i_ is the corresponding Mahalanobis distance. The line of equal probability between the presence and absence groups in multivariate space is now no longer linear, and thus, the technique is now described as non-linear discriminant analysis (NLDA).

(1) $$\mathrm{Pr}(1/x)=\frac{p_{1}|C_{1}{|}^{-1/2}e^{-D^{2}1}/2}{\sum_{g=1}^{2}p_{g}|C_{g}{|}^{-1/2}e^{-D^{2}g}/2}$$

$$\mathrm{Pr}(2/x)=\frac{p_{2}|C_{2}{|}^{-1/2}e^{-D^{2}2}/2}{\sum_{g=1}^{2}p_{g}|C_{g}{|}^{-1/2}e^{-D^{2}g}/2}$$

where $\left|C_{1}\right|$ and $\left|C_{2}\right|$ are the determinants of the covariance matrices for groups *g* = 1 and 2, respectively [34].

Another commonly encountered problem is that either the presence or absence cluster (or both) is not multivariate normal. To overcome this problem, the user of eRiskMapper is allowed to divide the observations into a number of clusters each for presence and absence observation using the k-means cluster algorithm [35]. This algorithm essentially makes a series of clusters for presence and absence observations, each of which is closer to multivariate normality than the original group from which those observations were drawn. Thus, clustering allows the requirements of NLDA to be met.

#### 2.3.2. Variable Selection

Identification of variables that are important in discriminating the presence and absence of any disease is a critical step in NLDA. eRiskMapper uses a forward step-wise procedure in which variables are included one at a time based on their ability to improve the discrimination of the groups. The discriminating criteria can be selected by the user from a small group, and these criteria include AIC, AIC_c_, F-test, Mahalanobis distance, AUC, and Kappa (the details of which are explained later). eRiskMapper automatically chooses up to ten variables in each model, although not all ten need to be used in fitting the data (for example, the AICc criterion may suggest a smaller number to avoid over-fitting).

#### 2.3.3. Variable Selection Criteria

In this study, the AICc (corrected Akaike Information Criteria) [36] was used as the selection criterion for including a variable through the forward step-wise method for eventual use in modeling. If the inclusion of an additional variable decreases the AICc by more than a threshold value (5 AIC_C_ units), then the variable is included; if it is less than the threshold value, then it is not included.

#### 2.3.4. Bootstrapping

Sparse datasets are very common in species’ and diseases’ distribution modeling. Bootstrapping is one of the methods that can be employed on sparse data and examines the likely importance of variability within the training set on overall model predictions. Thus, for example, if several bootstrapped models give very different results, the training set itself must be highly variable and it is therefore unlikely that it captures the full range of conditions in which the species occurs in nature. If, on the other hand, the bootstrapped models give more or less similar answers, then it is likely that the training set is representative of the range of conditions in nature. Thus, it is imagined that the relationship between reality and the training set is the same as that between the training set and its bootstrapped samples. There is no a priori knowledge of the nature of the first relationship, but this can be investigated by exploring the second relationship. In this study, 100 bootstraps were generated, and predictions were made for each bootstrap and combined to produce an average prediction: the final risk map. In each bootstrap, 200 points were selected from the presence set and 200 points for the absence (or pseudo-absence) set. Arranging samples to have equal number of presence and absence observations in each bootstrap produces model outputs with the greatest accuracy [37].

#### 2.3.5. Accuracy and Validation Statistics

Different accuracy statistics, such as sensitivity, specificity [38,39] or Kappa [40,41,42], were computed for each bootstrap model, and the average accuracy statistics across 100 bootstraps are presented. Sensitivity measures the proportion of actual presence sites predicted as presence, and specificity measures the proportion of actual absence sites predicted as absence. Kappa (k) is an index of agreement that is often used to assess model accuracy and varies between −1 (complete disagreement between predictions and observations) and 1.0 (complete agreement). Kappa = 0 when the predictions are not better than random (thus, some sites are predicted correctly). Kappa values < 0.4 indicate poor models, values between 0.4 and 0.75 indicate good models, and values greater than 0.75 indicate excellent models [40].

Other accuracy statistics such as AUC (Area under Curve) and Producer’s and Consumer’s Accuracies were also calculated for the 100 bootstrap models. The percentage of all known sites (both presence and absence) correctly classified by the model gives the Producer’s Accuracy and the Consumer’s Accuracy as the percentage of model predictions (presence and absence) that are correct. The Kappa, sensitivity, and specificity values were also calculated for the hold-out test data (out-of-sample) for each bootstrap, and the average validation statistics were calculated.

Three different NLDA models were run. In the first model (Model 1), the villages with no reported BTV outbreaks during the study period were taken as the absence sites; this model was run with one presence and two absence clusters. In the second model (Model 2), pseudo-absence data were generated and used as described earlier (Section 2.1), again with one presence and two absence clusters. Model 3 also used pseudo-absence data but fitted three presence and three absence clusters.

## 3. Results

The average accuracy statistics for the three models (Table 1) show that Model 1 was the worst model and Model 3 performed marginally better than Model 2 (Kappa was the same, but sensitivity and specificity were slightly higher). Similarly, the average validation statistics of Model 1 were poorest among the three models. Model 3′s Kappa value indicates a slightly worse fit than Model 2, but its sensitivity and specificity were marginally higher than Model 2 again. It should be emphasized that any single model provides only a snapshot of the information contributing to the overall final risk map, i.e., the average of 100 model outputs, each with a different bootstrap sample and each possibly with a different set of predictor variables.

### 3.1. Single Best Model Results

The mean values of the predictor variables in the best single Model 3 bootstrap model are shown in Table 2. The presence clusters (P1, P2, and P3) and absence clusters (A1, A2, and A3) are arranged row-wise, and the columns (1–10) represent the variable number as per their order of selection (an indication of their importance in the model). The last column (11) indicates the sample size of each cluster. Table 2 shows that BT occurs most commonly in areas with low values of variance and biannual amplitude of nighttime land surface temperature (variance = 9.18 vs. 12.76, biannual amplitude = 1.32 vs. 1.71, and biannual phase = 3.28 vs. 3.52) than in areas with more seasonally variable nighttime land surface temperatures. However, high values of the annual and tri-annual phases of nLST (annual = 5.23 vs. 5.18 and tri-annual phase = 1.25 vs. 1.13), i.e., later seasonal peaks of nighttime temperature favor BT transmission. The areas with high NDVI (mean 0.41 vs. 0.46) and EVI (mean = 0.26 vs. 0.28), reflective of dense forest environments, do not favor BT transmission.

Comparing the roles of different Fourier variables in each presence and absence cluster shows slightly different results from the mean values. For example, nLST variance is higher in P2 compared to A2 and A3 (variance = 11.0 in P2 vs. 7.5 and 3.0 in A2 and A3, respectively), which is in contrast to the overall means for the presence and absence sites for this variable (overall, nLST variance is lower in presence than in absence sites). Similarly, the tri-annual phase and annual phase of nLST are lower in P1, P2, and P3 compared to A2 and A3.

The model accuracy matrix (also called a ‘confusion matrix’) for the best model is shown in Table 3. The matrix shows the observed and predicted category classification. In one presence cluster (P1), there were 62 observed absences, and 52 were assigned to this category correctly, with only 2 observations assigned to the absence category. Likewise, out of a total of 200 presence points, 32 were misclassified (only 3 were misclassified in the absence category), and among the 200 absence points, only 11 points were misclassified.

The overall accuracy result of this best model is shown in Table 4. The Producer’s and Consumer’s Accuracies are very high (>80%) for all the categories, except for the presence 3 category, which had 67.4% Consumer’s Accuracy. In such a table, some misclassification errors are more serious than others. For example, from the Consumer’s point of view, an absence site belonging to one cluster but assigned to another absence cluster is a less serious error (since both clusters refer to absence) than one where an absence site is assigned to a presence cluster. If we combine all presence and all absence clusters before calculating these statistics, the overall Consumer’s Accuracy for this model is 96.6% for predicted presence sites and 98.5% for predicted absence sites.

### 3.2. Averaged Bootstrap Model Results

**Figure 1:**
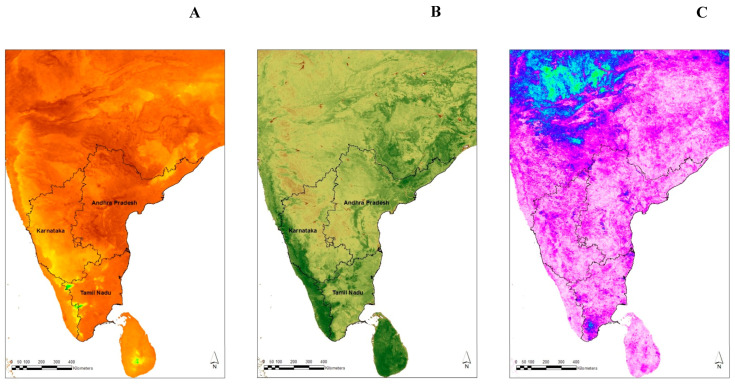
Maps of potential explanatory discriminating variables affecting the presence and absence of bluetongue in South India: (**A**) maximum daytime LST (the maximum temperature is less along the western region), (**B**) mean NDVI (the NDVI is high (darker green) along the Western Ghat region and in a few areas in Tamil Nadu and Central and North-Eastern Andhra Pradesh), and (**C**) tri-annual amplitude day time LST (the tri-annual amplitude of temperature is low (white to light pink color) along the western region and high in other regions (dark pink to blue) [26].

**Figure 2:**
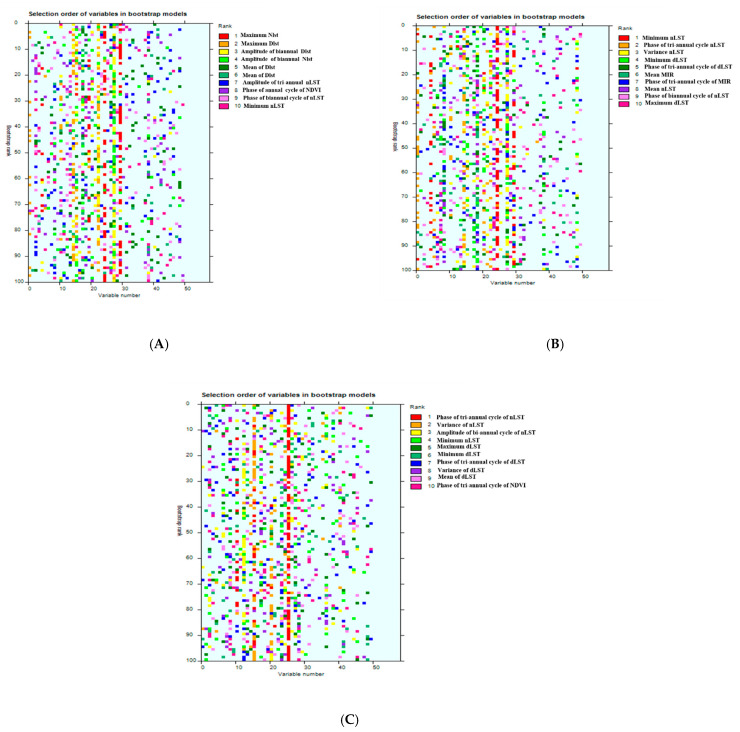
Graphical representation (‘rainbow plots’) of the ranks of predictor variables to show how often any particular variable was selected across the 100 bootstrap models for models 1 (**A**), 2 (**B**), and 3 (**C**). Each row in these figures refers to a single bootstrap model (arranged in rank order from 1 to 10, with the best model at the top), and each column refers to one of the predictor variables. In each model, the variables are color-coded on a rainbow color scale, i.e., the top predictor variable is shown in red, the second most important variable is shown in orange, and so on (hence the description ‘rainbow plot’ for such images). There is a predominant red line (phase of nLST tri-annual cycle) in Model 3, indicating not only that this variable was frequently selected, but also that it was often selected first in the various bootstrapped models. There are fewer signs of an overall dominant variable in the other two plots.

The important and selected variables are shown in Figure 1. The rainbow plots showing the relative importance of all predictors in all 100 models are shown in Figure 2. The top-ranked variable in Model 1 is the maximum nighttime land surface temperature, the minimum nighttime land surface temperature in Model 2, and the tri-annual phase of nighttime land surface temperature in Model 3. With the exception of one NDVI variable in Model 1 and Model 3 each, temperature variables were dominant in all the models.

**Figure 3:**
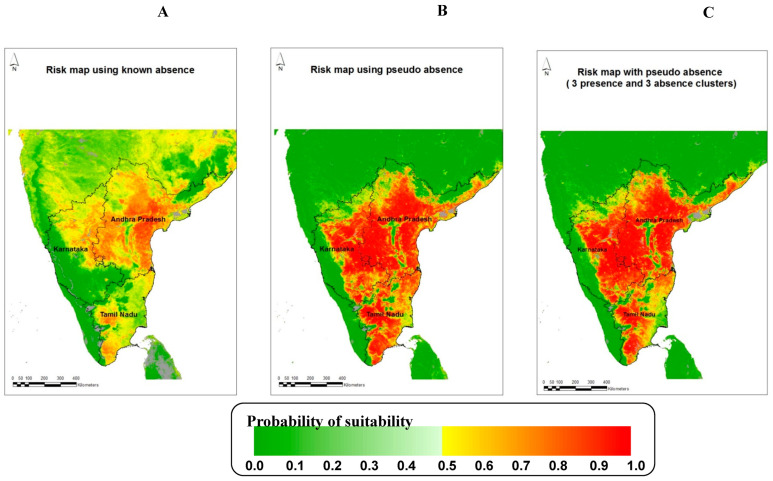
Risk map for bluetongue in South India using Fourier processed MODIS variables with (**A**) Model 1 (known absence with 1 presence and 2 absence clusters), (**B**) Model 2 (pseudo-absence with 1 presence and 2 absence clusters), and (**C**) Model 3 (pseudo-absence with 3 presence and 3 absence clusters). The probability of suitability is on a scale of zero to one. Probabilities from 0.0 to 0.49 are shown in green (darker to lighter green), indicating the predicted absence of disease. Probabilities from 0.50 to 1.0 are colored yellow to dark red, indicating conditions predicted as being suitable for bluetongue.

The final risk maps for the three different models (the average of 100 bootstrapped sample models in each case) are shown in Figure 3, and the top ten variables are presented in Table 5. The final risk map for Model 1 (Figure 3) is strikingly different from those of the other two models, indicating large areas of high risk, mainly in the state of Andhra Pradesh. The Model 2 and Model 3 sets of models, which were much more accurate in validation, predict larger areas of high risk across all three states but correctly predict low risks in the western areas (Western Ghat forest regions), northern parts of Karnataka, and northern and a few north-eastern and central parts of Andhra Pradesh (Eastern Ghat forest regions).

**Figure 4:**
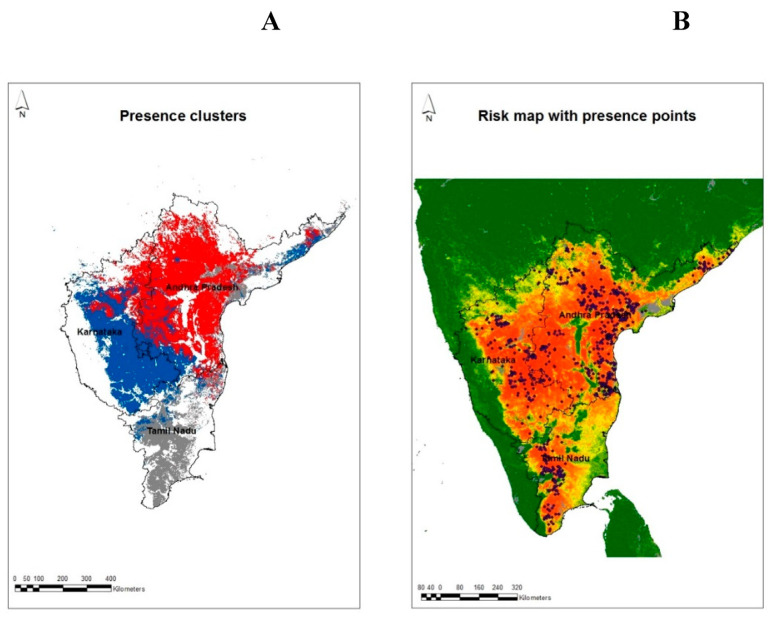
(**A**) Distribution of clusters in Model 3, with 3 presence and 3 absence clusters. The absence clusters are shown in white. Presence clusters are shown in red, blue and grey color. Notice that each cluster is relatively restricted geographically, one to each state. (**B**) Risk map developed with Model 3 with bluetongue presence points (blue dots).

The distribution of the three presence clusters for the best model in Model 3 is shown in Figure 4. Rather surprisingly, the clusters each seem restricted to a different state. Clusters 1 (red dots), 2 (blue), and 3 (grey) are mostly found in Andhra Pradesh, Karnataka, and Tamil Nadu, respectively. Such a restriction of clusters (based on environmental variables) to particular states indicates that the environmental conditions of those states consistently differ from each other. It is possible that such environmental conditions in turn drive differences in the epidemiology of BTV in the three states, potentially involving different key vector and/or host species or virus strains. This result also highlights the potential danger of extrapolating any model based on one state’s data to other, even adjacent states that may (and, in the present case, clearly do) experience different environmental conditions or social conditions such as agricultural practices.

## 4. Discussion

Although there is a high seroprevalence of BTV across the majority of states in India, regular outbreaks of BT are primarily reported in South India. To date, there have been no studies that have identified risk areas and factors for BT at the village level, with most studies concentrating on the molecular aspects of the virus or seroprevalence rather than the disease [43]. The district-level forecast system predicts the most likely occurrence of BT in districts 2 months in advance, and there is no risk map at the village level. Long warning times are required for the timely production of required vaccination doses. In this study, we developed a risk map generated using temporal Fourier transformed variables that are remotely sensed, which discriminates between the presence and absence areas with high model accuracy for both training and test data, and it may be useful for planning vaccination and surveillance in high-risk areas. Bluetongue has been reported every year since 1992 in South India [2]. Hence, a cost-benefit analysis needs to be carried out to know the economic benefits of vaccination against BT in high-risk areas identified in our study.

Overall, the model with pseudo-absence points and three presence and three absence clusters performed better than the other two models considered in this study. The model based on known absence points (villages that never recorded a BT outbreak) gave a poor fit compared to models including pseudo-absence points. The underlying reason for this result is not completely clear, but it may result from the underreporting of BT or spatially variable reporting efforts during outbreaks. The minimum distance rule used to generate pseudo-absence data means that absence sites are environmentally more different from any presence site than the absence villages are likely to have been in the original dataset—some of which may have been very close to presence villages, both geographically and environmentally. Selecting pseudo-absence sites in this way may artificially inflate the overall model accuracy (because absence sites are all very different from presence sites; hence, discriminating between the two sorts of sites will be easy), and this should also be considered when comparing models with different sorts of absence sites. The averaged bootstrap model accuracy (sensitivity = 0.97 and specificity = 0.96) is very high, and the validation statistics on “out-of-fit” test data (sensitivity = 0.88 and specificity = 0.91) are also very good considering the very different environmental conditions across the three states under study. Most of the models for predicting BT or *C. imicola* presence and absence in other countries have been validated internally, and the percentage of correct classification ranged from 75% to 95% [5,6,7,22].

In all three models, temperature variables were predominant in the highest-ranked variables (Table 3). The selection of temperature variables in discriminating presence and absence areas is as expected because temperature not only influences the different life stages of *Culicoides* but is also a major determinant of the extrinsic incubation period (the time taken for BTV to infect and replicate to transmissible levels in the vector) [16]. The higher values of nLST variance in the P2 cluster compared to A2 and A3 (Table 2) show the influence of temperature between the presence and absence groups, and therefore, comparing the overall means of a single variable of the presence and absence groups can be misleading. Instead, it is the unique combination of variables (not the value of any single one) in each group that tends to determine disease presence and absence. The diversity of environmental conditions may be a contributing factor to the presence of different epidemiological systems along with the diversity of hosts and breeds in South India, as identified in a previous study [2]. The detection of different groups is also supported by the fact that there are different *Culicoides* species identified in each state and different serotypes detected in the past [3]. However, studies examining the role of *Culicoides* species in transmitting different serotypes/strains are lacking. The presence of three distinct presence and absence groups (Figure 4A), which corresponds to the three states under study, is supported by the fact that major areas of these states fall under different agroecological zones of the country [44]. The present study also stresses the usefulness of Fourier variables in discriminating between the different areas of endemism of the disease. In addition, the seasonality of different potential vectors may vary in each state, and further studies are required to study the influence of environmental factors on the abundance of these vectors.

In other studies, NDVI variables were important in describing the distribution of *C. imicola* in temperate regions [21,22,45,46], and a study [47] on the risk of the Oropouche virus (OROV) in Central and South America was modeled. In one study [13], different variables were selected for each vector species modeled (NDVI for *C. pulicaris* and *C. imicola* and temperature variables for *C. obsoletus* group and *C. newsteadi*). However, NDVI, along with minimum LST, explained a greater variance (67%) compared to a model with only minimum LST (explaining only 40% of the variance) in a *C. imicola* abundance model [45]. In another study, the mean of middle infrared (MIR) reflectance was the most important variable in determining the presence of *C.imicola*, but NDVI was selected in an abundance model [23]. In the present study, NDVI is higher (Figure 1B) in areas where BT has never been reported (Figure 4B). The best risk map developed here shows that the areas along the Western Ghat (Figure 3C) are less suitable for BT transmission. This region is covered by forest and has fewer sheep and lower transportation of livestock from other regions of South India than other parts of the state. The Western Ghat regions have not reported BT outbreaks in the past twenty years ([2] and Figure 4B). The Eastern Ghat region is similar to the Western Ghat region in many respects and is also predicted by the model to be at a very low risk of BT outbreaks. There is a common belief among researchers and field veterinarians that BT is endemic to the entire region of South India, but both the data and the risk maps show that there are areas within each state that are very unlikely to experience BT outbreaks because the environmental conditions are unlike any of the sites from which BT has been recorded to date. Although there is seroprevalence of BT in Kerala [48], which forms part of the Western Ghat, no clinical outbreaks of BT have been reported in the past 20 years or so. One of the reasons for the absence of clinical disease may be due to the low sheep population and high goat population (goats are relatively resistant to BTV compared to sheep) or due to the host specificity of potential vectors in Kerala.

The map of the presence clusters shows that a considerable area is covered by each cluster, which is, nevertheless, relatively geographically distinct from the other clusters. This suggests that BTV transmission and impacts may be different in the different areas, perhaps involving different vectors or vector complexes, hosts, and agricultural management systems, creating different environments for transmission, or any combination of these effects. Thus, there is an urgent need for systematic vector and host ecology and competence studies in the region. In conclusion, discriminant analysis, incorporating different clusters of disease presence, sheds an interesting light on potentially different epidemiological situations and raises relatively precise questions for future studies to address.

Risk maps have been used for the planning prevention and control of many livestock diseases such as Rift Valley fever [49,50], rabies [51], and anthrax [52], among many other diseases. For anthrax, risk maps were useful in identifying the gaps in vaccine coverage in high-risk groups and risk areas [52] as well as informing efforts to target the livestock population [53]. Spatial analysis of avian influenza helped identify the spatial range over which the highly pathogenic avian influenza viruses spread between farms and can be used to control the outbreaks [54].

For BT, studies from around the world have utilized risk mapping to plan the prevention and control of the disease. The relative risk of the disease varies across geographical areas in Switzerland, and such tools were valuable in the evaluation of control programs for BT [55]. Early warning systems using GIS and transmission models were developed to predict the incursion of bluetongue in Switzerland and plan for the targeted surveillance of *Culicoides* [56]. A risk map for the occurrence of *C. imicola* was also developed in Italy and further validated by field collections of the vector [57]. The entomological surveillance data on *C. imicola* were then used to develop probability maps and to define the geographical risk of bluetongue [58]. The R0 was estimated for different months to identify the risk months for BTV for estimating the proportion of the population requiring vaccination to prevent BT outbreaks in Kazakhstan [59].

Inactivated BTV pentavalent vaccines are manufactured and supplied by Indian Immunologicals, but vaccination is not mandatory and is being carried out only in districts of South India to which BTV is endemic based on demand from farmers. There are also no guidelines by the state or central government for vaccination in high-risk areas using risk maps or on the basis of forecasting. The risk map developed in this study can be utilized to prioritize high-risk areas for vaccination and make better use of limited veterinary personnel. The village-level risk maps could be helpful to both district-level officers and veterinary officers in planning vaccination in higher-risk areas on a priority basis and in creating awareness among farmers. Targeted vaccination at the village level will not only help in controlling the disease but could also minimize the wastage of vaccines.

Risk maps require dissemination and co-development with stakeholders for planning their vaccination and surveillance strategies. The risk maps can be disseminated in the form of leaflets or maps to all high-risk areas to prevent the disease. Village-level predictions can be provided to the district-level officers in a table format (list of villages), who in turn can inform the village-level veterinary officers to carry out vaccinations and awareness campaigns in high-risk areas. The risk maps can also be provided on a website for the stakeholders, along with instructions on how to use such maps and predictions. The risk maps and predictions can also be used by the researchers and epidemiologists working on bluetongue to plan surveillance and vector surveys. The interaction between the stakeholders, epidemiologists, and researchers at regular intervals can help improve the risk maps and predictions.

The risk map developed in this study can be utilized by policymakers for systematic vaccination in high-risk areas. Currently, there is no vaccination strategy for BT. The vaccination is carried out using pentavalent (containing five serotypes) vaccines by sheep farmers, and it is not part of the vaccination guidelines in South India. The yearly vaccination, if carried out, in high-risk areas identified in the risk map developed in this study can prevent losses for subsistence sheep farmers. However, the risk map needs to be updated with new data as the risk of bluetongue may change in the future due to changes in climate, land use, and agriculture [60].

## Figures and Tables

**Table 1:** Accuracy and validation accuracy statistics (Kappa, sensitivity, and specificity) for the three models. See text for the conventional interpretation of the Kappa values. Models 2 and 3 have excellent performance on the training set compared to model 1. Model 1 performed poorly on the validation set compared to Models 2 and 3. Overall, Model 3 performed marginally better than Model 2.

	Kappa	Sensitivity	Specificity
Model 1			
Accuracy statistics	0.54 ± 0.048	0.79 ± 0.033	0.74 ± 0.037
Validation accuracy statistics	0.20 ± 0.039	0.69 ± 0.094	0.66 ± 0.027
Model 2			
Accuracy statistics	0.84 ± 0.036	0.93 ± 0.02	0.94 ± 0.014
Validation accuracy statistics	0.67 ± 0.063	0.87 ± 0.062	0.90 ± 0.011
Model 3			
Accuracy statistics	0.84 ± 0.026	0.97 ± 0.013	0.96 ± 0.013
Validation accuracy statistics	0.64 ± 0.035	0.88 ± 0.069	0.91 ± 0.012

**Table 2:** Mean values of the top ten ranked variables from the best bluetongue model with the lowest AIC_C_ among the 100 bootstrap models. The mean values for the top ten variables for three presence (upper rows) and three absence (lower rows) clusters. The last two columns show the mean values for presence (P) and absence (A), respectively. See Table 3 for the key to variable names and mean model ranks.

	nLST Variance	Biannual Amplitude of nLST	Minimum dLST	Biannual Phase of nLST	Mean EVI	Tri-Annual Phase of Nlst	Annual Phase of nLST	Mean NDVI	Variance of Dlst	Mean of dLST	Sample Size
P1	7.27	1.5	303.05	3.26	0.24	1.26	4.87	0.39	21.90	308.09	62
P2	11.0	1.31	303.66	3.32	0.25	1.25	5.35	0.40	33.36	310.18	103
P3	7.2	1.03	301.08	3.21	0.30	1.23	5.52	0.47	26.97	308.42	35
A1	15.56	1.95	301.73	3.54	0.24	0.96	5.32	0.41	45.26	309.27	144
A2	7.5	1.22	298.82	3.34	0.34	1.65	5.66	0.54	20.25	304.61	32
A3	3	0.87	297.54	3.62	0.45	1.47	3.74	0.70	9.37	301.11	24
Mean P	9.18	1.32	303.02	3.28	0.26	1.25	5.23	0.41	28.69	309.22	200
Mean A	12.76	1.71	300.76	3.52	0.28	1.13	5.18	0.46	36.95	307.54	200

**Table 3:** Mean ranks of the top ten variables from all the 100 bootstrap models in Models 1, 2, and 3.

	Top Ten Variables in Model 1	Top Ten Variables in Model 2	Top Ten Variables in Model 3
1	Maximum nLST	Minimum nLST	Phase of tri-annual cycle of nLST
2	Maximum dLST	Phase of tri-annual cycle nLST	Variance of nLST
3	amplitude of biannual dLST	Variance nLST	Amplitude of biannual cycle of nLST
4	Amplitude of biannual nLST	Minimum dLST	Minimum nLST
5	Mean of Dlst	Phase of tri-annual cycle of dLST	Maximum dLST
6	Amplitude of tri-annual nLST	Mean MIR	Minimum dLST
7	Phase of biannual cycle of dLST	Phase of tri-annual cycle of MIR	Phase of tri-annual cycle of dLST
8	Phase of annual cycle of NDVI	Mean nLST	Variance of dLST
9	Phase of biannual cycle of nLST	Phase of biannual cycle of nLST	Mean of dLST
10	Minimum nLST	Maximum dLST	Phase of tri-annual cycle of NDVI

**Table 4:** Accuracy matrix of the best model (pseudo-absence with 3 presence and absence clusters each) among 100 bootstrap models. Accuracy matrix of the best model (pseudo-absence with 3 presence and absence clusters each) among 100 bootstrap models. The observed categories are in rows, and the predicted categories in the column. For a perfect model fit, all the numbers should be on the diagonal, with no off-diagonal entries. P1–P3 are the presence categories, and A1–A3 are the absence categories.

		Predicted Category						
Observed category		P1	P2	P3	A1	A2	A3	Tot.
	P1	52	5	3	0	2	0	62
	P2	9	87	7	0	0	0	103
	P3	1	4	29	0	1	0	35
	A1	1	1	0	139	2	1	144
	A2	0	1	4	0	27	0	32
	A3	0	0	0	0	1	23	24
	Tot.	63	98	43	139	33	24	400

**Table 5:** Overall accuracy statistic for the best model among 100 bootstrap models. The overall Kappa accuracy of this model is 0.86, and its AUC is 0.9987.

Category	%Correct	%Producer’s Accuracy	%Consumer’s Accuracy
P1	83.87	83.87	82.54
P2	84.47	84.47	88.78
P3	82.86	82.86	67.44
A1	96.53	96.53	100
A2	84.38	84.38	81.82
A3	95.83	95.83	95.83

## Data Availability

The risk maps developed in this study are provided in the main manuscript. Restrictions apply to the availability of raw bluetongue outbreak data. Data were obtained from departments of Animal husbandry of Karnataka, Andhra Pradesh, and Tamil Nadu and are available from the authors with the permission of the data providers.
